# Synthesis of uniform cyclodextrin thioethers to transport hydrophobic drugs

**DOI:** 10.3762/bjoc.10.310

**Published:** 2014-12-09

**Authors:** Lisa F Becker, Dennis H Schwarz, Gerhard Wenz

**Affiliations:** 1 Organic Macromolecular Chemistry, Saarland University, Campus C4.2, 66123 Saarbrücken, Germany

**Keywords:** active pharmaceutical ingredient, binding constant, cyclodextrin, derivatization, gas chromatography, sevoflurane, substitution pattern

## Abstract

Methyl and ethyl thioether groups were introduced at all primary positions of α-, β-, and γ-cyclodextrin by nucleophilic displacement reactions starting from the corresponding per-(6-deoxy-6-bromo)cyclodextrins. Further modification of all 2-OH positions by etherification with iodo terminated triethylene glycol monomethyl ether (and tetraethylene glycol monomethyl ether, respectively) furnished water-soluble hosts. Especially the β-cyclodextrin derivatives exhibit very high binding potentials towards the anaesthetic drugs sevoflurane and halothane. Since the resulting inclusion compounds are highly soluble in water at temperatures ≤37 °C they are good candidates for new aqueous dosage forms which would avoid inhalation anaesthesia.

## Introduction

Cyclodextrins (CDs) are cyclic oligomers of α-1,4-linked glucose units. Those CDs consisting of 6, 7, and 8 glucose units are called α-, β- and γ-CD, respectively [1]. CDs are well known to increase the bioavailability of active pharmaceutical ingredients (APIs) [2–3], and they are readily available in pharmaceutical purity and industrial quantities. Furthermore, they are water soluble and regarded as non-toxic in case of α- and γ-CD [4–5], while β-CD shows some toxic effects such as haemolysis at high concentrations [6].

CDs are generally employed to increase the bioavailability of those APIs scarcely soluble in water [7]. The observed solubilization of an API is generally based on the complexation of the hydrophobic part of the API molecule within the CD cavity [3]. There are several formulations of APIs containing CDs on the market, such as prostaglandine/α-CD [8], and piroxicam/β-CD [9].

Further application of native CDs for the delivery of hydrophobic drugs is often hampered by aggregation [10], and generally by poor solubility of the formed inclusion compounds. As a consequence, the phase solubility isotherm shows saturation behaviour, so-called B-type curves [11–12]. Therefore many CD derivatives have been synthesized to overcome this problem [1,12–13]. Statistical β-CD derivatives, such as hydroxypropyl-β-CD [14], methylated β-CD [15], and sulfobutyl-β-CD [16] are indeed in use, but quality control for such mixtures of compounds remains a difficult issue [17–18]. Also binding potentials of statistical β-CD derivatives like hydroxypropyl-β-CD are often smaller than those of native β-CD [19].

We recently found out that full methylation of all secondary hydroxy groups of β-CD causes a significant drop of binding potential, while substitution at the primary site does not alter the binding potential or even increases it [20]. Especially substitution of all primary hydroxy groups by thioether groups gives rise to compounds with very high binding potentials due to the higher hydrophobicity of sulfur compared to oxygen [21–23]. The octa-substituted carboxyethyl thioether of γ-CD is already in use under the name sugammadex (Bridion^®^) for the reversal of neuromuscular blockade, making use of its extremely high affinity towards rocuronium [24–25]. Furthermore, hydrophilic γ-CD thioethers show high affinities to other guests such as polycyclic aromatic hydrocarbons [26], botulin [27–28], and fullerene C_60_ [29].

Hydrophilic β-CD thioethers also tightly complex volatile benzene derivatives leading to a significant decrease of their vapour pressure [30]. Therefore we were encouraged to investigate the inclusion of volatile hydrophobic APIs, e.g., sevoflurane, in CD thioethers. Sevoflurane, a versatile inhalational anaesthetic [31], was already included in native CDs and hydroxypropyl-β-CD, but these complexes are either nearly insoluble in water or the binding constants are rather low [32–34].

We focussed our effort on the design of hydrophilic and/or amphiphilic CD thioethers, because only amphiphilic molecules can form [35–36] or incorporate into bilayer membranes [37–38]. Amphiphilic CD carriers can enter a bilayer membrane to support the API to overcome cellular barriers, such as the intestinal barrier [39] or the blood-brain barrier (BBB) [40]. Long alkyl chains (C_4_–C_12_) have already been attached via thioether or sulfoxide linkages to all primary positions by Kawabata and Ling et al. to form hydrophobic β-CD derivatives [41–42]. Mazzaglia et al. reported on amphiphilic β-CD derivatives with alkyl chains (C_2_–C_16_) connected by the thioether linkages to the primary site and a statistical substitution with oligoethylene glycol at secondary sites [43]. Becker et al. describe similar hosts with 2,2,2-trifluoroethyl groups at all primary sites and also oligoethylene glycol at secondary sites [44]. In both latter cases statistical CD derivatives have been employed, where both the lengths of the oligoethylene oxide side chains and their locations were scattered.

**Scheme 1:**
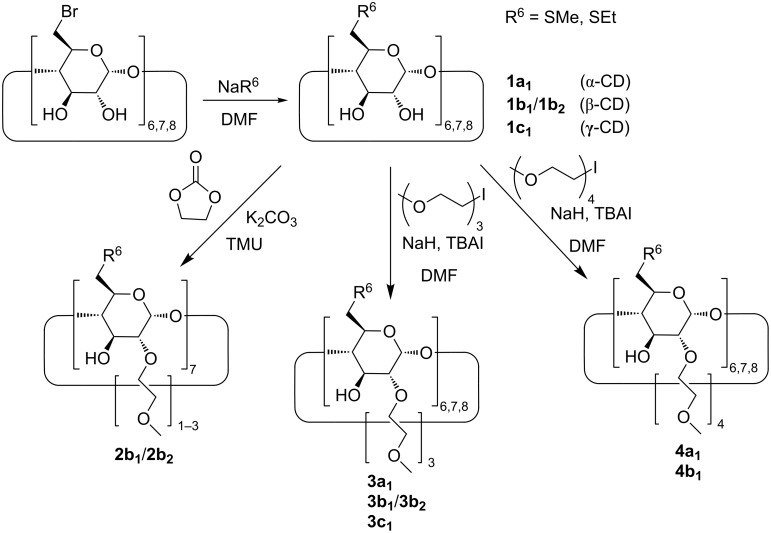
Synthetic route to neutral water-soluble CD thioethers.

Herein, we report on the synthesis of water soluble CD derivatives (Scheme 1) with well-defined molecular structure and high binding affinities towards volatile anaesthetic APIs.

## Results and Discussion

**Figure 1:**
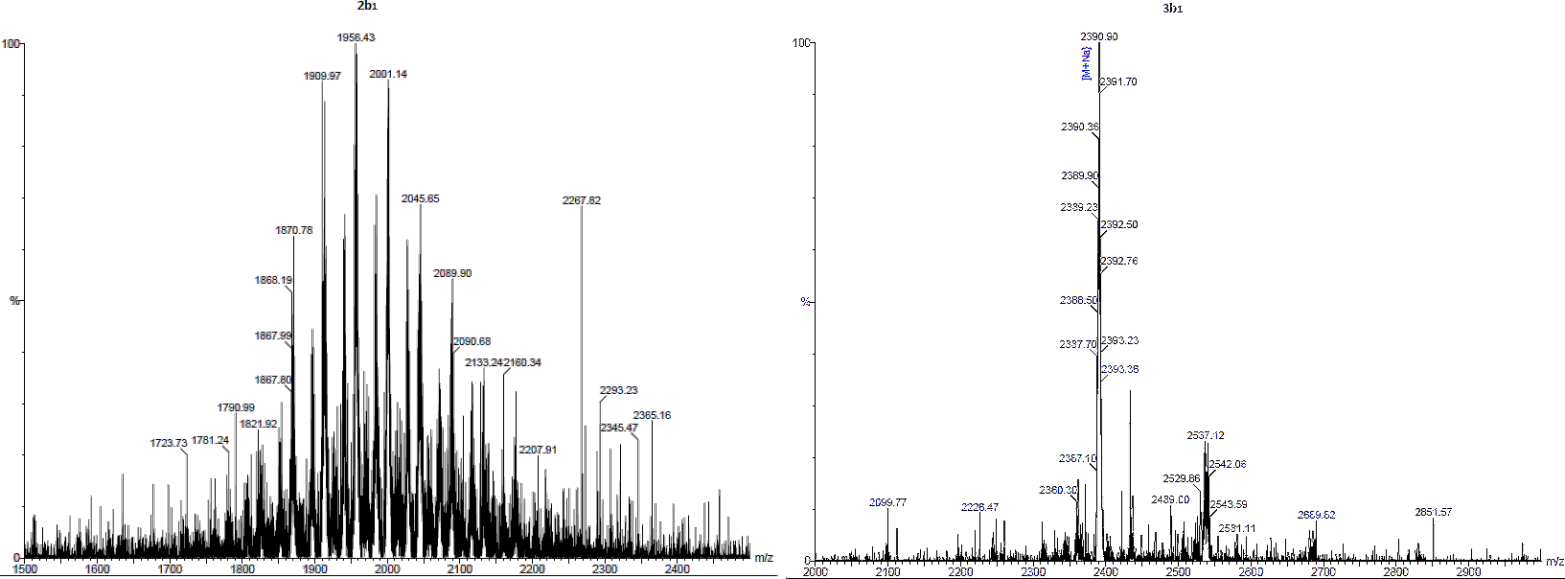
ESI MS spectra of CD derivatives **2b_1_** (left) and **3b_1_** (right).

**Figure 2:**
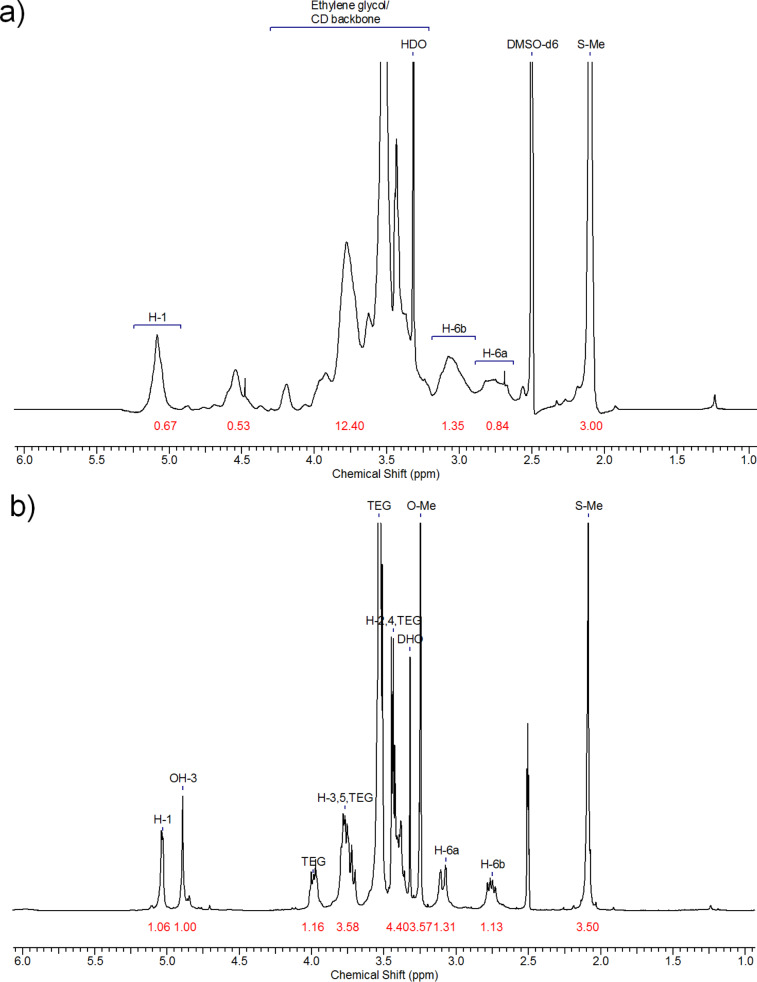
^1^H NMR spectra of a) the statistical CD derivatives **2b_1_** and b) the corresponding uniform derivative **3b_1_** in DMSO-*d*_6_ (numbers in red are the integrals of the respective signals).

Heptakis-6-deoxy-6-bromo-β-CD, synthesized according to Defaye et al., was reacted with sodium methanethiolate, and ethanethiolate, respectively [45]. The reaction was performed in DMF solution leading to the corresponding thioethers **1b_1_**/**1b_2_** in excellent yields (up to 92%). Afterwards, these thioethers **1b_1_**/**1b_2_** were hydroxyethylated with ethylene carbonate to the water soluble derivatives **2b_1_**/**2b_2_** according to Mazzaglia et al. [43]. The ESI MS of **2b_1_** (Figure 1) showed a rather broad molecular weight distribution typical for CD derivatives with statistical substitution pattern. On the other hand, nearly uniform CD derivatives were synthesized by regioselective deprotonation of all 2-OH positions with NaH in DMF solution according to Tian and D’Souza [46], and subsequent complete alkylation with I-(CH_2_-CH_2_-O)*_n_*-CH_3_ (*n* = 3,4) for 4–7 d at 60–80 °C. The resulting derivatives **3** and **4** were isolated by liquid–liquid extraction at 50 °C with a Kutscher–Steudel extractor and subsequent column chromatography. Yields were high as shown in Table 1. The ESI MS of **3b_1_** (Figure 1) showed a significantly lower polydispersity than **2b_1_**. Also the ^1^H NMR spectrum of **3b_1_** was much better resolved than the one of the statistical derivative **2b_1_** due to its homogenous substitution pattern and uniform lengths of the oligoethylene oxide groups (Figure 2).

**Table 1:** List of the synthesized CD derivatives and their lower critical solution temperatures (LCST).

	Ring size	R^6^	R^2^	Yield [%]	LCST [°C]

**3a_1_**	6	SMe	(CH_2_CH_2_O)_3_Me	42%	43
**4a_1_**	6	SMe	(CH_2_CH_2_O)_4_Me	10%^a^	65
**3b_1_**	7	SMe	(CH_2_CH_2_O)_3_Me	68%	42
**3b_2_**	7	SEt	(CH_2_CH_2_O)_3_Me	89%	61
**4b_1_**	7	SMe	(CH_2_CH_2_O)_4_Me	14%^a^	54
**3c_1_**	8	SMe	(CH_2_CH_2_O)_3_Me	89%	49

^a^Loss of compound during ultrafiltration.

**Figure 3:**
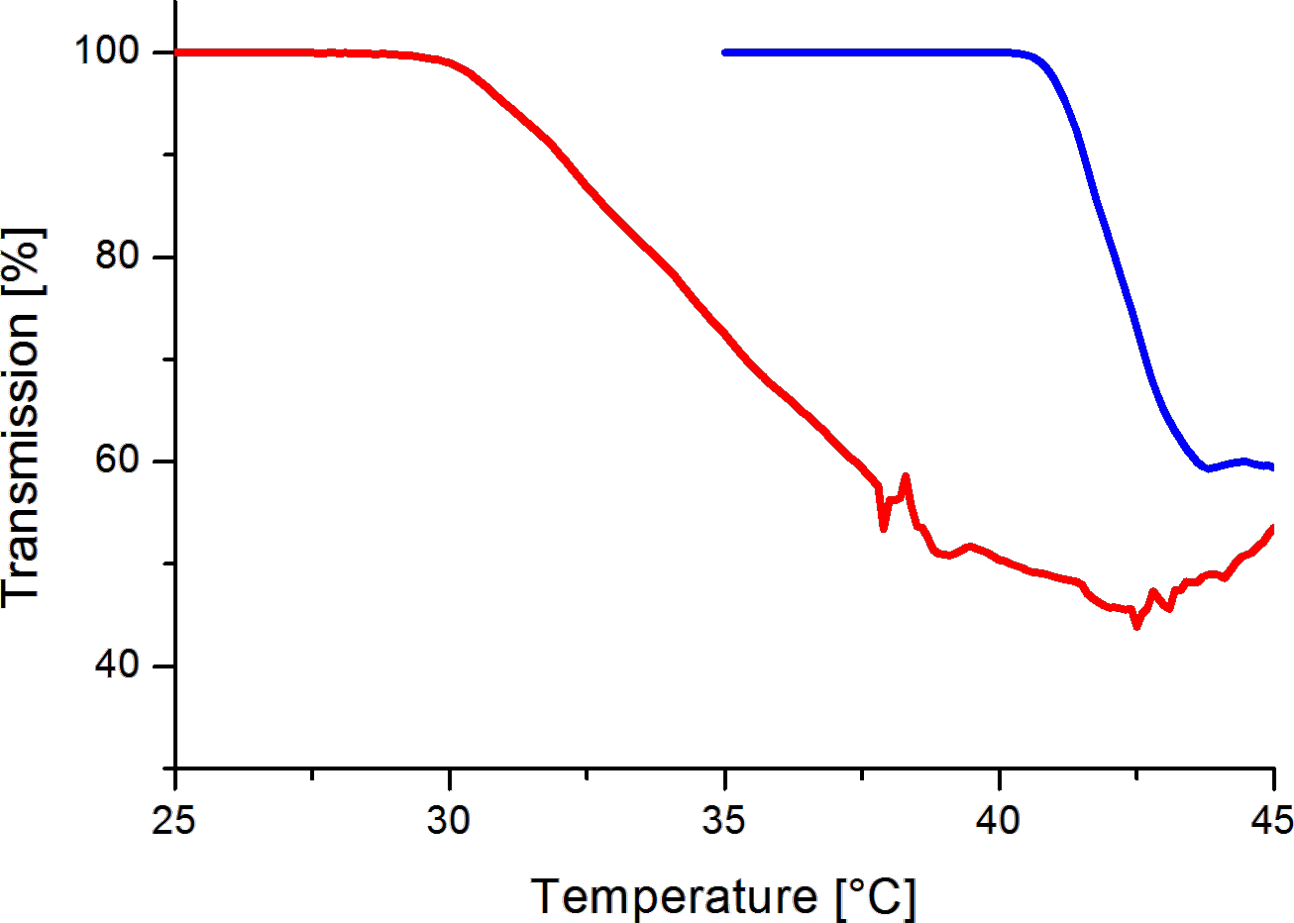
Transmission (λ = 670 nm) of aqueous solutions (1.0 wt %) of **2b_1_** (red) and **3b_1_** (blue).

All β-CD derivatives **2**, **3** and **4** were indeed highly soluble in water at 25 °C but upon heating the clear solutions turned turbid at a certain temperature and the compounds precipitated. The observed phase separation at the so-called lower critical solution temperature (LCST) is typical for uncharged polymeric amphiphiles, such as methyl cellulose [47], poly(*N*-isopropylacrylamide) (pNiPAAm) [48], and also for methylated CDs [49], and CDs completely modified with oligoethylene glycol units [50]. While the LCST transition of the statistical derivative **2b_1_** was within a rather broad temperature range (30–40 °C), the uniform derivative **3b_1_** showed a sharp transition at 42 °C (Figure 3). The LCST was only scarcely dependent on the ring size of CD but increased with the length of the hydrophilic oligoethylene oxide chain, as listed in Table 1. The LCST should be beyond 40 °C for being applicable for the delivery of drugs into a mammalian body.

### Investigation of the inclusion of sevoflurane

**Figure 4:**
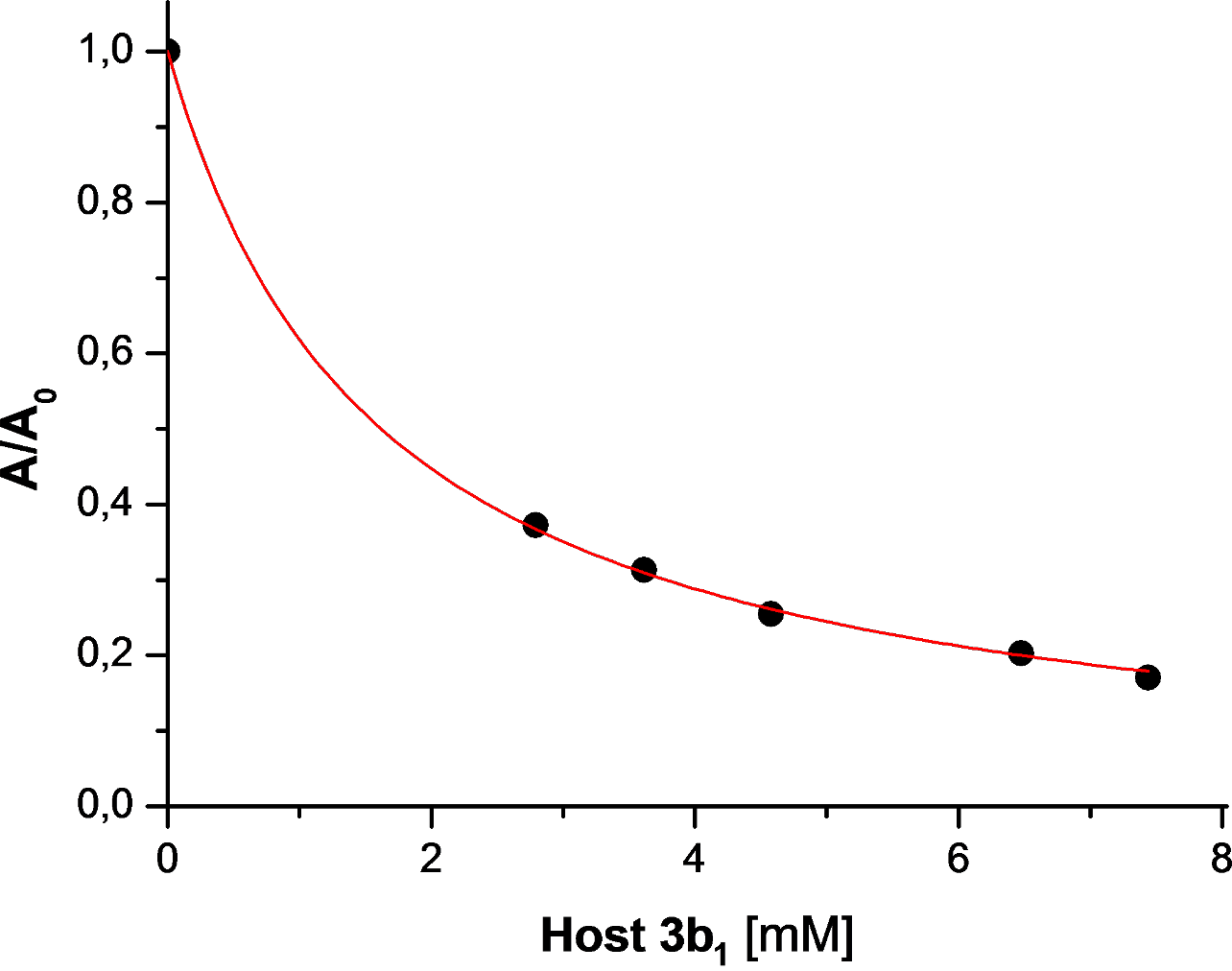
Decay of the relative vapour pressure *A*/*A_0_* as function of the host concentration **3b_1_** measured by GC headspace; the curve was fitted according to Equation 1.

The inclusion of the anaesthetic sevoflurane by our hosts was investigated by the measurement of the vapour pressure of the guest by gas chromatography as a function of the host concentration as described by Armstrong [51] and Fourmentin et al. [30,52]. As shown in Figure 4, the vapour pressure of the guest sevoflurane significantly drops due to complexation by host **3b_1_**.

The corresponding binding constant *K* was calculated from the hyperbolic decay of the area *A* of the sevoflurane signal with the total concentration of the CD derivative [CD]_0_ by non-linear regression according to Equation 1, as described previously in this journal [30]. The Henry constant was determined according to a known GC method [30] to *k*_H_ = 2.0 at 25 °C and *k*_H_ = 3.05 at 37 °C in good agreement with literature data [53]. The occupancy *x* of employed CD host by the guest was calculated by the law of mass action according to Equation 2. The solubility of the free guest sevoflurane in water [*G*] = 5.4 mM at 25 °C was calculated by the ideal gas law from its Henry constant and vapour pressure *p* = 263 mbar [54–55], according to Equation 3:

[1]
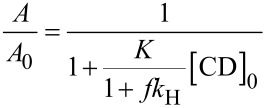


[2]
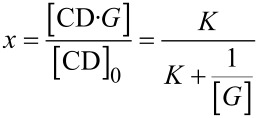


[3]
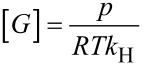


Commercially available native CDs and β-CD derivatives showed rather poor affinities to sevoflurane, as shown in Table 2. Among the native CDs β-CD had far the highest binding constant which was attributed to the best space filling of this host by sevoflurane. We recently demonstrated also for other guests that space filling has a very significant influence on the binding constants [30]. We found indeed an even higher binding constant for heptakis-2,6-di-*O*-methyl-β-CD (DIMEB), but medical applications remain questionable for this host because of its known high toxicity [6]. On the other hand, heptakis-2,3,6-tri-*O*-methyl-β-CD (TRIMEB) and the less toxic derivative hydroxypropyl-β-CD performed much worse. The low binding potential of TRIMEB was already found for other guests and can be attributed to the lack of intramolecular hydrogen bonds stabilizing the CD framework.

**Table 2:** Binding data for sevoflurane in native CDs and commercial CD derivatives at 25 °C.

Host	*K* [L/mol]	Occupancy *x* [mol %]

α-CD	18	9
β-CD	150	45
γ-CD	9	5
DIMEB	713	79
TRIMEB	27	13
HP-β-CD	163	47

The new hydroxyethylated CD thioethers, listed in Table 3, generally showed higher binding constants than the respective native CDs. The higher binding potential of CD thioethers was already found for other guests as well [21–23]. The binding constants of the α-CD derivatives **3a_1_** and **4a_1_** were much lower than the ones of the corresponding β-CD derivatives **3b_1_** and **4b_1_** which can be again rationalized by the better space filling of the seven membered rings by sevoflurane. The binding constant decreased with increasing lengths of the alkyl substituents at the sulfur atoms as well as with the lengths of the oligoethylene oxide chains. This fact was attributed to an increasing loss of entropy upon complexation of the guests. The longer the substituents the higher the conformational freedom of the host leading to higher intrinsic entropy. Also the two statistical derivatives, **2b_1_** and **2b_2_** showed somewhat lower binding constants than the regioselectively modified derivatives **3**, which might be due to a smaller amount of residual secondary hydroxy groups known to stabilize the CD framework by intramolecular hydrogen bonds [20]. Among the CD thioethers **3b_1_** performed best reaching occupancies close to 100%.

**Table 3:** Binding data for sevoflurane in the new CD thioethers at 25 °C.

Host	*K* [L/mol]	Occupancy *x* [mol %]

**3a_1_**	64	26
**4a_1_**	9	5
**2b_1_**	2270	92
**2b_2_**	263	59
**3b_1_**	2801	94
**3b_2_**	286	61
**4b_1_**	722	80

Although all binding measurements were already performed under physiological pH and ionic strength, we were interested in the binding potential of the best host **3b_1_** approaching in vivo conditions to estimate the performance of this CD derivative for the delivery in the bodies of animals or humans. As anticipated, the binding constant slightly dropped in 5 wt % albumin solution and further dropped in human serum (Table 4). At 37 °C a further decrease of *K* was observed, but it still remained rather high. The occupancy of **3b_1_** was still 87 mol % in human serum at body temperature. Therefore this compound should be well suitable for the delivery of sevoflurane. Potentially oral aqueous dosage forms can be developed for both anaesthesia and the treatment of pain. **3b_1_** is also able to complex other hydrofluoric anaesthetics, like halothane [54], where the binding constant *K* = 9090 L/mol (occupancy of the host 98%) was even higher than for sevoflurane.

**Table 4:** Binding data for sevoflurane in **3b_1_** for various media and temperatures.

Medium	Temperature [°C]	*K* [L/mol]	Occupancy *x* [mol %]

albumin^a^	25	2175	92
human serum	25	1802	90
water	37	1427	88
albumin^a^	37	1382	88
human serum	37	1331	87

^a^5 wt %.

## Conclusion

The water-soluble β-CD thioether **3b_1_** with the smallest possible substituents at both the sides of the CD torus showed the highest binding affinities for the anaesthetic APIs sevoflurane and halothane, much higher than native CDs and known CD derivatives. Since the LCST is sufficiently higher than body temperature, **3b_1_** is a very promising candidate for oral or intravenous delivery of these anaesthetics.

## Experimental

**Methods.** Characterization of all products was operated using NMR and ESIMS spectroscopy. All NMR spectra including ^1^H, ^13^C, H,H-COSY and C,H-COSY were measured at room temperature by a BrukerBioSpin spectrometer Magnet System 400 MHz Ultra shield plus (^1^H: 400 MHz, ^13^C: 100.6 MHz). The chemical shifts are given in parts per million (ppm) in relation to the corresponding solvent signal. The data analysis was performed with SpecManager included in ACDLabs 10.0 from Advanced Chemistry Development Inc., Toronto, Ontario, Canada*.* The proton and carbon atoms of the glucose units were marked with 1, 2, 3 etc. starting from the anomeric proton/carbon. The multiplicities were assigned as follows: s for singlet, d for doublet, t for triplet, bs for broad signal and m for multiplet. Mass spectra were recorded by a LC–MS spectrometer ZQ-4000 from Waters GmbH, Eschborn, Germany, operated in ESI^+^ and ESI^−^ mode.

Some products were purified by cross-flow nanofiltration using a membrane called Mini Mate TPP Capsule from Pall, Crailsheim, Germany, further a membrane called Omega with a Cut-off of 650 Da was used. Freeze-drying was carried out with a lyophilizer Lyophille Alpha 1–4 produced by Christ, Osterode am Harz, Germany. The LCST transitions were recorded with a UV–vis spectrometer Evolution 220 from Thermo Scientific, Waltham, MA, USA, equipped with a heating device from Harrick, Pleasantville, New York. The inclusion properties of the host molecules were investigated by head space gas chromatography with a Shimadzu GC-17A GC equipped with a head space unit from Shimadzu, Kyoto, Japan*.* Vials of 5 mL volume were used, the ratio between gas (*V* = 3.2 mL) and aqueous (*V* = 1.8 mL) phase was f = 1.77.

**Materials.** All chemicals (except CDs) were purchased from Sigma-Aldrich, Merck, Acros Organics, Fisher Scientific or TCI Europe and were used without further purification. α-, β- and γ-CD were kindly provided by Wacker Chemie AG, Munich, Germany and were used after drying overnight at 60 °C under reduced pressure. Human serum was kindly provided by University Hospital of Würzburg. All measurements were performed in saline HEPES-buffer solution (pH 7.4) with a NaCl concentration of 0.9 wt %.

**3b_1_: Heptakis[6-deoxy-6-methylsulfanyl-2-(2-(2-(2-methoxyethoxy)ethoxy)ethyl)]-β-cyclodextrin:**


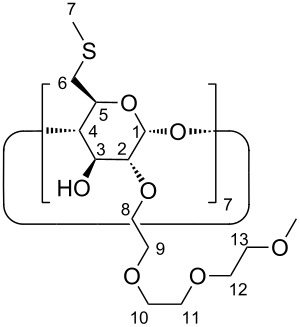


2.60 g (65 mmol) NaH (60 wt % dispersion in mineral oil, Sigma-Aldrich) was washed twice with 25 mL of *n*-pentane under N_2_ and stirred at rt for 1 h. After addition of 6.25 g (4.64 mmol) heptakis(6-deoxy-6-methylsulfanyl)-β-cyclodextrin dissolved in 130 mL of DMF, 17.8 g (65 mmol) 2-(2-(2-methoxyethoxy)ethoxy)ethyl iodide and 17.5 mg (0.05 mmol) tetra-*n*-butylammonium iodide were added and the resulting reaction mixture was stirred at 60 °C under N_2_ for 6 d. The reaction was quenched by the addition of 50 mL of ethanol and stirred at rt for further 30 min. The solvents were completely removed by vacuum distillation (bath temperature 70 °C, 1 mbar) and the residue was dissolved in 200 mL of water and neutralized by addition of 1 M HCl. The crude product was isolated by extraction with ethyl acetate at 70 °C using a Kutscher–Steudel extractor. The organic phase was concentrated in vacuo and the remaining residue was fractionized by column chromatography over 1.0 kg of silica (60 Å, 70–230 mesh, Fluka) with an ethyl acetate/methanol gradient (100/0 → 90/10 → 0/100 v/v) as eluent. The product (7.5 g, 68%) was obtained as a yellowish oil after complete removal of the eluent by vacuum distillation and drying at 60 °C in vacuo (0.03 mbar) for 3 d. TLC: *R*_f_ (EtOAc/MeOH 9:1 v/v) = 0.06; *R*_f_ (MeOH) = 0.57; ^1^H NMR δ/ppm (DMSO-*d*_6_, 400 MHz) 5.03 (d, *^3^J* = 3.3 Hz, 1H, H-1), 4.89 (s, 1H, OH-3), 4.01–3.96 (m, 1H, H-8a), 3.79–3.69 (m, 3H, H-3, H-5, H-8b), 3.53 (s, 8H, H-8, H-9) 3.50 (m, 1H, H-4), 3.44–3.38 (m, 3H, H-2, H-9), 3.24 (s, 3H, O-CH_3_), 3.10–3.07 (m, 1H, H-6a), 2.75 (dd, *^3^J* = 14.1 Hz, 7.8 Hz, 1H, H-6b), 2.08 (s, 3H, H-7); ^13^C NMR δ/ppm (DMSO-*d*_6_, 100 MHz) 100.5 (C-1), 85.5 (C-4), 71.3 (C-2, C-3, C-5), 69.8–69.6 (C-8, C-9), 58.0 (C-10), 35.0 (C-6), 16.0 (C-7); ESIMS *m*/*z*: 2390.90 [M + Na]^+^.

## Supporting Information

File 1 Experimental procedures for CD derivatives **1b_2_**, **3a_1_**, **4a_1_**, **3b_2_**, **4b_1_**, and **3c_1_**.
