# Supplementary material for: Synthesis of uniform cyclodextrin thioethers to transport hydrophobic drugs
Source: Beilstein J Org Chem. 2014 Dec 9;10:2920–7. doi: 10.3762/bjoc.10.310 (PMC4273231; doi:10.3762/bjoc.10.310)
Supplement: File 1 — Experimental procedures for CD derivatives 1b2, 3a1, 4a1, 3b2, 4b1, and 3c1. [file Beilstein_J_Org_Chem-10-2920-s001.pdf]

Supporting Information
for
Synthesis of uniform cyclodextrin thioethers to transport hydrophobic drugs

Lisa F. Becker, Dennis H. Schwarz and Gerhard Wenz*

Address: Organic Macromolecular Chemistry, Saarland University, Campus C4.2, 66123 Saarbrücken, Germany

* Corresponding author

Email: Gerhard Wenz - g.wenz@mx.uni-saarland.de

Experimental procedures for CD derivatives $1b_2$, $3a_1$, $4a_1$, $3b_2$, $4b_1$, and $3c_1$

$1b_2$: Heptakis(6-deoxy-6-ethylsulfanyl)-β-cyclodextrin

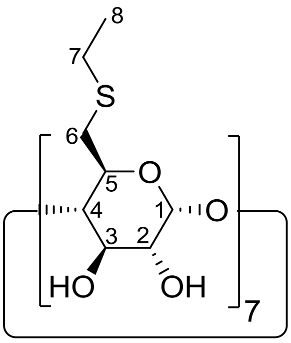

4.00 g (2.54 mmol) heptakis-(6-deoxy-6-bromo)-β-cyclodextrin was added to a solution of 100 mL DMF and 4.49 g (53.38 mmol, 21 equiv) sodium ethanethiolate

under vigorously stirring. The reddish brown solution is stirred for another 120 h at 90 °C. After cooling down to rt, the product is precipitated in 1.3 L of ice water and stirred for another 2.5 h. A white solid (3.15 g, 86%) is obtained after filtration and drying in vacuo.

^{1}H-NMR:	δ/ppm (DMSO-d_6, 400 MHz) = 1.17 (dd, 3H, H-8, ^{2}J=4,8 Hz, ^{3}J=6 Hz), 2.59 (q, 2H, H-7, ^{2}J=4,8 Hz, ^{3}J=12 Hz), 2.78-2.83 (dd, 1H, H-6b, ^{2}J=8 Hz, ^{3}J=12 Hz), 3.09 (d, 1H, H-6a, ^{3}J=8 Hz), 3.35-3.37 (m, 2H, H-2,4), 3.61 (dd, 1H, H-3, $^{2/3}J$=8 Hz,), 3.76-3.79 (m, 1H, H-5), 4.89 (d, 1H, H-1, ^{3}J=4 Hz), 5.86 (bs, 1H, OH-3), 5.94 (bs, 1H, OH-2).
^{13}C-NMR:	δ/ppm (DMSO-d_6, 100 MHz) = 14.9 (C-8), 26.3 (C-7), 32.9 (C-6), 71.4 (C-5), 72.3 (C-2), 72.6 (C-3), 84.9 (C-4), 102.0 (C-1).
MS(ESI):	m/z= 1465.43 $[M+Na]^{+}$.
CHN:	for $C_{56}H_{98}O_{28}S_7$ (M = 1443.82 g/mol)
	calculated: C (46.58 %), H (6.84 %)
	found: C (47.22 %), H (6.81 %).

3a$_1$: Hexakis[6-deoxy-6-methylsulfanyl-2-(2-(2-(2-methoxyethoxy)ethoxy)ethyl)]-*α*-cyclodextrin

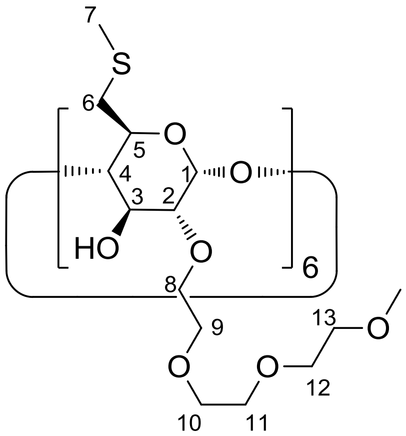

0.82 g (20.5 mmol) NaH (60 wt % dispersion in mineral oil, Sigma-Aldrich) was washed twice with 10 mL of *n*-pentane under N_2 and stirred at rt for 1 h. After addition of 1.96 g (1.7 mmol) hexakis(6-deoxy-6-methylsulfanyl)-*α*-CD dissolved in 50 mL of DMF, 5.62 g (20.5 mmol) 2-(2-(2-methoxyethoxy)ethoxy)ethyl iodide and 7 mg (0.016 mmol) tetra-*n*-butylammonium iodide were added and the resulting reaction mixture was stirred at 60 °C under N_2 for 6 d. The reaction was quenched by addition of 10 mL of methanol and stirred at rt for further 30 min. The solvents were completely removed by vacuum distillation (bath temperature 70 °C, 1 mbar) and the residue was dissolved in 40 mL of water and neutralized by addition of 1 M HCl. The crude product was purified by nanofiltration in water (500 Da, Nadir PM NP030, Microdyn-Nadir GmbH, Wiesbaden, Germany) and a yellowish oil (1.44 g, 42%) was obtained after lyophilization.

^{1}H-NMR δ/ppm (DMSO-d_6, 400 MHz) = 5.00 (bs, 1H, H-1), 4.61 (bs, 1H, OH-3), 3.89-3.95 (m, 1H, H-8a), 3.78-3.80 (m, 1H, H-5), 3.81 – 3.86 (m, 1H, H-3), 3.71-3.77 (m, 1H, H-8b), 3.50-3.55 (m, 10H, H-10, 11, 12, 13) 3.43-3.45 (m, 1H, H-4), 3.41 – 3.42 (m, 2H, H-9), 3.34 – 3.37 (m, 1H, H-2), 3.23 (s, 3H, O-CH_3), 3.05-3.08 (m, 1H, H-6a), 2.74-2.79 (m, 1H, H-6b), 2.07 (s, 3H, H-7).

^{13}C-NMR: δ/ppm (DMSO-d_6, 100 MHz) = 100.2 (C-1), 85.9 (C-4), 80.2 (C-2), 72.8 (C-3), 71.3 (C-9), 70.8 (C-8) 70.7 (C-5), 69.5-69.9 (C-10, C-11, C-12, C-13), 58.1 (O-CH_3), 35.1 (C-6), 16.2 (C-7).

4a$_1$: Hexakis[6-deoxy-6-methylsulfanyl-2-(2-(2-(2-(2-methoxyethoxy)ethoxy)ethoxy)ethyl)]-*α*-cyclodextrin

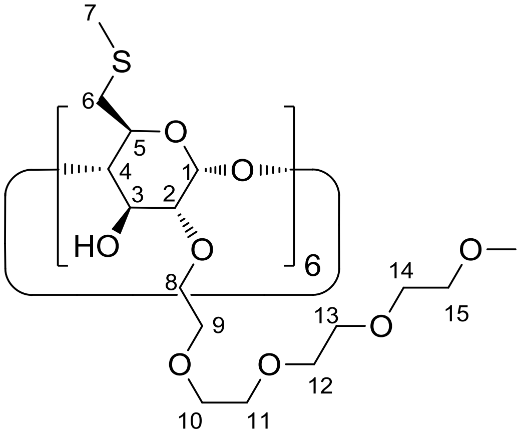

0.42 g (10.4 mmol) NaH (60 wt % dispersion in mineral oil, Sigma-Aldrich) was washed twice with 10 mL of *n*-pentane under N_2 and stirred at rt for 1 h. After addition of 1.00 g (0.87 mmol) hexakis(6-deoxy-6-methylsulfanyl)-α-cyclodextrin dissolved in 50 mL of DMF, 2.25 g (10.4 mmol) 2-(2-(2-(2-methoxyethoxy)ethoxy)ethoxy)ethyl iodide and 3 mg (0.008 mmol) tetra-*n*-butylammonium iodide were added and the resulting reaction mixture was stirred at 80 °C under N_2 for 7 d. The reaction was quenched by addition of 10 mL of methanol and stirred at rt for further 30 min. The solvents were completely removed by vacuum distillation (bath temperature 70 °C, 1 mbar) and the residue was dissolved in 50 mL of water and neutralized by addition of 1 M HCl. The crude product was purified by crossflow nanofilration in water (1 kDa, Pall Minimate TFF Capsule) and a brown yellowish oil (0.204 g, 10%) was obtained after lyophilization.

TLC: R_f (EE:^{i}PrOH:NH_4OH:H_2O 7:7:5:2 v/v) = 0.58.

^{1}H-NMR δ/ppm (DMSO-d$_6$, 400 MHz) = 5.02 (d, 1H, H-1, ^3J= 3.0 Hz), 4.64 (s, 1H, OH-3), 3.98-3.94 (m, 1H, H-8a), 3.85-3.81 (m, 1H, H-3), 3.81-3.77 (m, 1H, H-5), 3.77-3.74 (m, 1H, H-8b), 3.53-3.52 (m, 12H, H-9, H-10, H-11, H-12, H-13, H-14), 3.45-3.42 (m, 2H, H-15), 3.36-3.35 (m, 1H, H-2),

3.25 (s, 3H, O-CH_3), 3.10-3.07 (m, 1H, H-6a), 2.81-2.75 (m, 1H, H-6b), 2.09 (s, 3H, H-7).

^{13}C-NMR: δ/ppm (DMSO-d_6, 100 MHz) = 100.2 (C-1), 85.9 (C-4), 80.1 (C-2), 72.8 (C-3), 71.3 (C-9), 70.8 (C-8), 70.7 (C-5), 69.8-69.6 (C-10, C-11, C-12, C-13, C-14, C-15), 58.1 (O-CH_3), 35.2 (C-6), 16.2 (C-7).

ESI-MS: m/z= 2316.49 $[M+Na]^+$.

3b$_2$: Heptakis[6-deoxy-6-ethylsulfanyl-2-(2-(2-(2-methoxyethoxy)ethoxy)ethyl)]-β-cyclodextrin

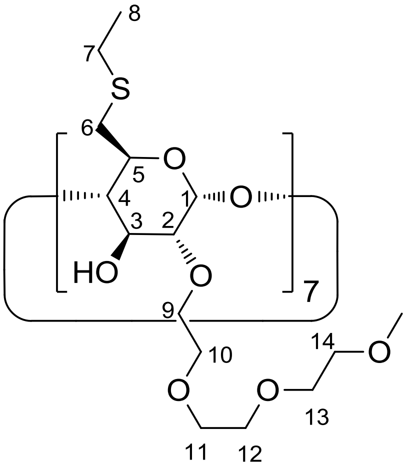

0.358 g (9.70 mmol) NaH (60 wt % dispersion in mineral oil, Sigma-Aldrich) was washed twice with 10 mL of *n*-pentane under N_2 and stirred at rt for 1 h. After addition of 1.00 g (0.69 mmol) heptakis(6-deoxy-6-methylsulfanyl)-β-CD dissolved in 40 mL of DMF, 2.66 g (9.70mmol) 2-(2-(2-methoxyethoxy)ethoxy)ethyl iodide and 3 mg (0.008 mmol) tetra-*n*-butylammonium iodide were added and the resulting reaction mixture was stirred at rt under N_2 for 3 d. The reaction was quenched by addition of 10 mL of methanol and stirred at rt for further 30 min. The solvents were completely removed by vacuum distillation (bath temperature 70 °C, 1 mbar) and the residue was dissolved in 40 mL of water and neutralized by addition of 1 M HCl. The crude product

was purified by crossflow nanofilration in water (1kDa, Pall Minimate TFF Capsule) and a yellowish oil (1.51 g, 89%) was obtained after lyophilization.

^{1}H-NMR δ/ppm (DMSO-d_6, 400 MHz) = 5.03 (bs, 1H, H-1), 4.87 (bs, 1H, OH-3), 3.97-3.99 (m, 1H, H-9a), 3.72-3.78 (m, 3H, -CH_2-CH_2-O), 3.50 – 3.53 (m, 8H, -CH_2-CH_2-O), 3.39 – 3.44 (m, 4H, -CH_2-CH_2-O), 3.24 (s, 3H, O-CH_3), 3.03-3.06 (m, 1H, H-6a), 2.82-2.86 (m, 1H, H-6b), 2.58 (q, 2H, H-7), 1,63 (dd, 3H, H-8).

^{13}C-NMR: δ/ppm (DMSO-d_6, 100 MHz) = 100.4 (C-1), 85.1 (C-4), 80.6 (C-2), 72.5 (C-3), 71.3 (C-10), 71.1 (C-9) 70.7 (C-5), 69.6-69.9 (C-11, C-12, C-13, C-14), 58.1 (O-CH_3), 32.7 (C-6), 26.4 (C-7), 14.8 (C-8).

ESI-MS: m/z= 2489.00 $[M+Na]^+$.

4b$_1$: Heptakis[6-deoxy-6-methylsulfanyl-2-(2-(2-(2-(2-methoxyethoxy)ethoxy)ethoxy)ethyl)]-*β*-cyclodextrin

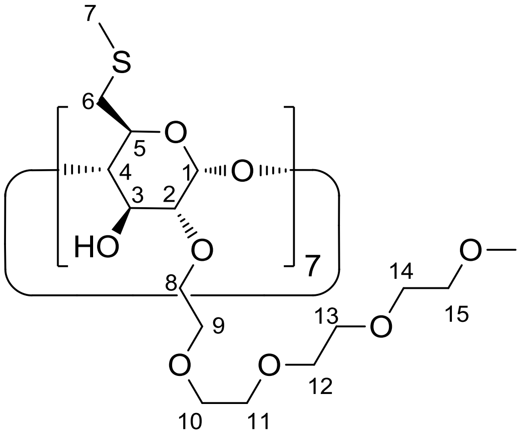

0.36 g (8.92 mmol) NaH (60 wt % dispersion in mineral oil, Sigma-Aldrich) was washed twice with 10 mL of *n*-pentane under N_2 and stirred at rt for 1 h. After addition of 1.01 g (0.74 mmol) heptakis(6-deoxy-6-methylsulfanyl)-β-cyclodextrin dissolved in 50 mL of DMF, 2.84 g (8.92 mmol) 2-(2-(2-(2-methoxyethoxy)ethoxy)ethoxy)ethyl

iodide and 3 mg (0.008 mmol) tetra-*n*-butylammonium iodide were added and the resulting reaction mixture was stirred at 80 °C under N_2 for 7 d. The reaction was quenched by addition of 10 mL of methanol and stirred at rt for further 30 min. The solvents were completely removed by vacuum distillation (bath temperature 70 °C, 1 mbar) and the residue was dissolved in 50 mL of water and neutralized by addition of 1 M HCl. The crude product was purified by crossflow nanofilration in water (1 kDa, Pall Minimate TFF Capsule) and a brown yellowish oil (0.277 g, 14%) was obtained after lyophilization.

^{1}H-NMR δ/ppm (DMSO-d_6, 400 MHz) = 5.03 (d, 1H, H-1, ^3J= 3.0 Hz), 4.89 (s, 1H, OH-3), 4.00-3.94 (m, 1H, H-8a), 3.78-3.70 (m, 1H, H-8b), 3.53-3.50 (m, 12H, H-9, H-10, H-11, H-12, H-13, H-14), 3.44-3.38 (m, 4H, H-2, H-4, H-15), 3.24 (s, 3H, O-CH_3), 3.10-3.06 (m, 1H, H-6a), 2.78-2.69 (m, 1H, H-6b), 2.08 (s, 3H, H-7).

^{13}C-NMR: δ/ppm (DMSO-d_6, 100 MHz) = 100.6 (C-1), 85.6 (C-4), 80.6 (C-2), 72.6 (C-3), 71.3 (C-9), 70.8 (C-8), 70.7 (C-5), 69.8-69.6 (C-10, C-11, C-12, C-13, C-14, C-15), 58.1 (O-CH_3), 35.1 (C-6), 16.0 (C-7).

ESI-MS: m/z= 2699.55 $[M+Na]^+$.

$3c_1$: Octakis[6-deoxy-6-methylsulfanyl-2-(2-(2-(2-methoxyethoxy)ethoxy)ethyl)]-*γ*-cyclodextrin

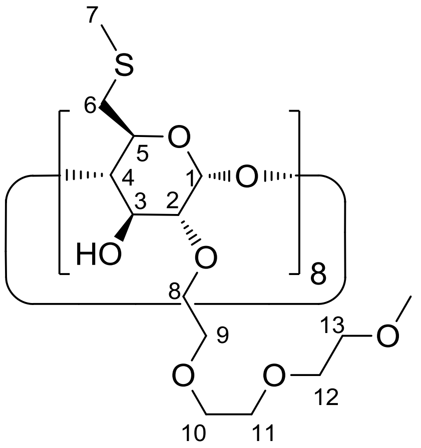

0.418 g (10.4 mmol) NaH (60 wt % dispersion in mineral oil, Sigma-Aldrich) was washed twice with 10 mL of *n*-pentane under N_2 and stirred at r.t. for 1 h. After addition of 1.01 g (0.65 mmol) octakis(6-deoxy-6-methylsulfanyl)-*γ*-cyclodextrin dissolved in 50 mL of DMF, 2.90 g (10.6 mmol) 2-(2-(2-methoxyethoxy)ethoxy)ethyl iodide and 3 mg (0.008 mmol) tetra-*n*-butylammonium iodide were added and the resulting reaction mixture was stirred at 60°C under N_2 for 4 d. The reaction was quenched by addition of 10 mL of methanol and stirred at r.t. for further 30 min. The solvents were completely removed by vacuum distillation (bath temperature 70 °C, 1 mbar) and the residue was dissolved in 50 mL of water and neutralized by addition of 1 M HCl. The crude product was purified by crossflow nanofilration in water (1 kDa, Pall Minimate TFF Capsule) and a yellowish oil (1.57 g, 89%) was obtained after lyophilization.

TLC: R_f (EE:^{i}PrOH:NH_4OH:H_2O 7:7:5:2 v/v) = 0.82.

^{1}H-NMR δ/ppm (DMSO-d_6, 400 MHz) = 5.10 (d, 1H, H-1, ^3J =3.0 Hz), 4.84 (bs, 1H, OH-3), 3.92-3.96 (m, 1H, H-8a), 3.76-3.80 (m, 1H, H-8b), 3.73-3.76 (m, 1H, H-5) 3.68-3.70 (m, 1H, H-3), 3.50-3.53 (m, 8H, H-10, 11, 12, 13)

3.41-3.44 (m, 1H, H-9), 3.38–3.41 (m, 2H, H-2), 3.35–3.38 (m, 1H, H-4), 3.23 (s, 3H, O-CH_3), 3.05-3.10 (m, 1H, H-6a), 2.71-2.76 (m, 1H, H-6b), 2.09 (s, 3H, H-7).

^{13}C-NMR: δ/ppm (DMSO-d_6, 100 MHz) = 99.8 (C-1), 84.1 (C-4), 80.7 (C-2), 72.3 (C-3), 71.3 (C-9), 71.0 (C-8) 70.7 (C-5), 69.5-69.9 (C-10, C-11, C-12, C-13), 58.0 (O-CH_3), 35.1 (C-6), 16.1 (C-7).

ESI-MS: m/z= 2729.32 $[M+Na]^+$.
